# Impairment in the Intention Formation and Execution Phases of Prospective Memory in Parkinson's Disease

**DOI:** 10.3389/fnins.2018.00098

**Published:** 2018-02-23

**Authors:** Shu-Hong Jia, Kai Li, Wen Su, Shu-Hua Li, Hai-Bo Chen

**Affiliations:** Department of Neurology, Beijing Hospital, National Center of Gerontology, Beijing, China

**Keywords:** Parkinson's disease, prospective memory, executive function, neuropsychological assessment, cognitive impairment

## Abstract

**Objective:** Patients with Parkinson's disease have prospective memory impairments. However, little is known about distinct phases of prospective memory in these patients. This study was designed to elucidate the specific phase(s) of prospective memory that are impaired in patients with Parkinson's disease.

**Methods:** The study included 31 Parkinson's disease patients and 27 healthy controls. The four phases of prospective memory (intention formation, retention, initiation, and execution) were examined in a complex prospective memory task. In this task, the participants were asked to form a sophisticated plan for performing six subtasks to obtain the highest score, and then execute the plan following a cue embedded in a questionnaire. Global cognitive function and relevant cognitive abilities, including attention, short-term memory, working memory, and inhibition, were also evaluated during the retention phase of the prospective memory task.

**Results:** Intention formation was impaired in Parkinson's disease patients (*p* < 0.001 vs. healthy controls). This impairment could not be attributed to deficits in other cognitive functions. The score of intention execution was also lower in Parkinson's disease patients (*p* = 0.004 vs. healthy controls). Such a difference was related to working memory deficits in Parkinson's disease. The intention retention and initiation were intact in Parkinson's disease patients. The score of intention execution correlated negatively with disease severity and disease duration.

**Conclusions:** Prospective memory in Parkinson's disease patients is impaired at the phase of intention formation. The worsening performance of intention execution in Parkinson's disease may be related to working memory deficits. In addition, prospective memory impairment might progress with increasing disease duration and severity.

## Introduction

Prospective memory (PM) is defined as remembering to perform intended actions in the future. It is critical for everyday life, and a deficit can result in severe consequences (Kliegel and Martin, 2003; Altgassen et al., 2007). PM can be classified into event-based PM (EBPM) and time-based PM (TBPM), depending on the type of cues. EBPM is remembering to execute an action when some external event occurs, while TBPM is remembering to perform a task at a certain time (Einstein and McDaniel, 1990). As a complex cognitive process, PM is divided into four phases: intention formation, intention retention, intention initiation, and intention execution (Kliegel et al., 2011). The prefrontal cortex plays a super-ordinate role during all the four phases of PM (Burgess et al., 2011). Thus, in addition to retrospective memory, PM cognitive processing requires a range of different executive functions in the prefrontal lobe, including planning, working memory, attention, strategizing, inhibition, mental flexibility, and task switching (Kliegel et al., 2011).

Cognitive impairments are common in Parkinson's disease (PD) and can affect the patients' quality of life (Leroi et al., 2012). Considering the importance of the prefrontal-striatal circuit for PM and cognitive impairments of PD, it is reasonable to speculate that patients with PD have PM impairments. Katai et al. (2003) found impaired EBPM in patients with PD, and their study suggested that impairment of PM in PD patients was not the result of forgetting the content of the PM, but the failure to retrieve it spontaneously. Some recent studies also demonstrated EBPM impairment in PD, possibly as a result of impaired self-retrieval processes (Pagni et al., 2011; Costa et al., 2013). In addition, TBPM has been reported to be impaired in PD, and this impairment is correlated with deficits in executive function and working memory (Costa et al., 2008b; Raskin et al., 2011).

Despite the above studies investigating PM among PD patients, the potential mechanism of PM impairment in PD is still unclear. An important approach to exploring the mechanism of PM utilizes an approach of focusing on the multiple phases of the PM process and the involvement of different cognitive resources (such as retrospective memory and executive functions) (Kliegel et al., 2011). Kliegel et al. (2005) used a complex PM task (a modified six-element task) to examine distinct phases of PM in PD patients. That study revealed impaired intention formation and a trend toward impairment in intention initiation in PD patients and suggested that there is an association between impairment in intention formation and working memory capacity deficits. The ambiguous results in intention initiation impairment might be attributed to the relatively small sample size (*n* = 16) in their study. More importantly, the absence of impairment in intention execution is not convincing considering the small sample size and also technical limitations. For example, Kliegel et al. (2005) only scored intention execution fidelity and the number of subtask shifts. Additionally, the requirement that the subjects write down the answers made motor function a confounding factor. This was particularly important because the patients had not taken their dopaminergic drugs for more than 12 h prior to the task. Previous studies have demonstrated that levodopa was associated with improved PM performance in PD patients (Costa et al., 2008a), making it clinically relevant to investigate the PM function without discontinuing dopaminergic medications.

Based on these previous studies, we hypothesized that PD patients might have impairments in intention formation, initiation, and the execution phases of PM, and that these impairments were associated with deficits of executive function, especially working memory. To test our hypothesis, we modified the paradigm of Kliegel et al., and investigated the different phases of PM in in a larger sample of PD patients, as well as exploring for additional influencing factors.

## Methods

### Participants

The current study included 31 patients with PD and 27 healthy control subjects. The two groups had comparable ages, sex distributions, global cognitive function, and education levels. The PD diagnoses for the subjects in the experimental group were made using the UK PD society brain bank clinical diagnostic criteria (Hughes et al., 1992) as verified by at least two experienced neurologists. Exclusion criteria included: (1) suspected dementia on the basis of clinical examination or a Mini-Mental State Examination (MMSE) score ≤ 24; (2) history or current evidence of major depressive disorder, anxiety, or psychosis; (3) history of head injury, stroke, or other neurological condition other than PD; (4) use of central nervous system therapies other than dopaminergic drugs; (5) presence of severe metabolic or systemic diseases. Thirty of the 31 patients were on dopaminergic treatments (e.g., levodopa, pramipexole, or piribedil, but not trihexyphenidyl). The mean levodopa equivalent daily dose was 457 mg, with a standard deviation of 288 mg. No patients were taking anticholinergic drugs. The PD patients continued their regular medication regimens and were tested 1–3 h after taking medication when they felt the dopaminergic drugs had taken effect (“on” state). The Hamilton Rating Scale for Depression (HRSD) was used to evaluate depression. All healthy controls had normal MMSE score and no history of neurological or psychiatric diseases. This study was approved by the institutional review board of Beijing Hospital and was conducted according to the Declaration of Helsinki. All participants gave written informed consent.

### Procedure

#### PM examination

**Figure 1:**
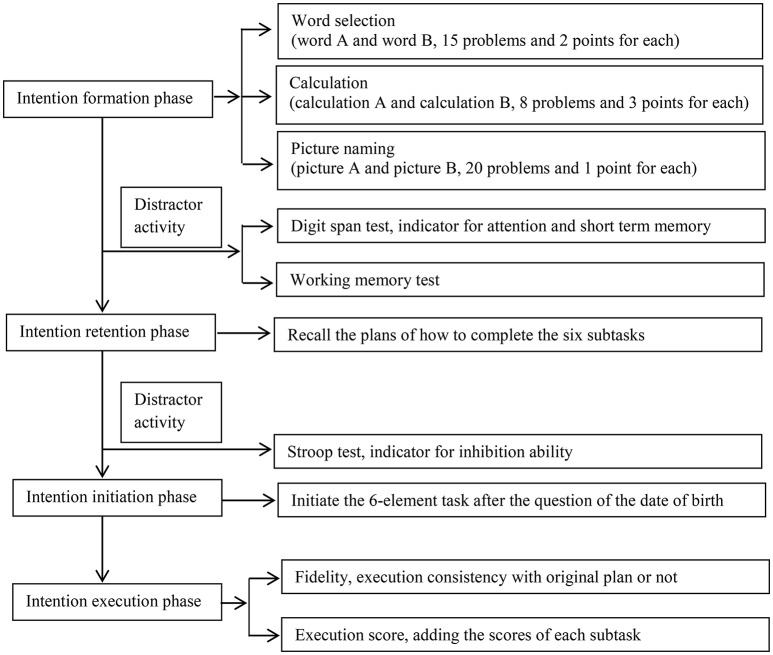
The flow chart of our procedure.

The test paradigm was modified from Kliegel et al. (2005). Briefly, the task consisted of 3 steps: (1) the intention formation phase, during which the participants were asked to make a plan on how to execute six subtasks according to a set of rules; (2) the intention retention phase, during which the participants completed the digit span test and the working memory test first, and then were required to recall their plan, and then took the Stroop test and had a 5-min rest; and (3) the intention initiation and execution phases, during which they should initiate and execute the plan upon seeing a cue embedded in a questionnaire (Figure 1). There were 3 modifications in the present paradigm compared with the one used by Kliegel et al. (2005), and we will explain them in detail in the following passages.

##### Intention formation phase

At the start of the experimental trial, the entire process of the task was explained to the participants in detail, so that they comprehended and remembered the instructions. They were asked to form a plan on executing the six subtasks. The subjects were instructed that, at a certain point in this test, they would be asked to fill out a personal information questionnaire, and they should start executing the plan (of the six subtasks) after answering a question (in the questionnaire) about their date of birth.

The six subtasks consisted of two similar sets (A and B) of three distinct types of tasks (word selection, calculation, and picture naming). The two sets were equivalent in difficulty level. The word selection task included 15 problems, and in each problem, the subjects were asked to point out the word that was not in the same category as the three remaining words. Each set of calculations consisted of 8 problems (e.g., 300/6 × 4). In the picture-naming task, each set had 20 pictures of common objects (e.g., a chair, table, or watermelon), and the subjects were required to vocally name the objects. The participants were required to complete the task verbally and not use writing (modification 1).

After explaining the six subtasks to the participants, we told the participants the following three rules on how they should arrange the order of each subtask, as well as, how performance was evaluated.

#### Rules

#1. The goal was to achieve the highest possible total score in executing the six subtasks. The scoring system was as follows: (1) The points earned in the earlier subtasks performed in each type (word selection, calculation, or picture naming tests) were doubled. (2) Errors and omissions were penalized by deductions of equal points. (3) Two points were awarded for finding a right word, 3 points for solving a calculation problem, and 1 point for naming a picture correctly (modification 2). The scoring system was modified from the Kliegel et al. (2005) study, in which the three kinds of subtasks were given equal weight. This modification was designed to incentivize the participants to produce more sophisticated plans than the even scoring system.
#2. Two subsets (A and B) of the same kind of subtask (word selection, calculation, or picture naming) could not be performed in succession. For example, a sequence of word A, word B, calculation A, calculation B, picture A, and picture B would represent a violation of this rule.
#3. Participants had only 6 min to complete these subtasks.

The participants studied the rules until they could accurately recall each of them. The score on the intention formation phase represented the sophistication of their planning. The total score in this phase was the sum of three components: (1) the number of the rules included in subject's intention (1 point for each rule); (2) whether the subject specified the sequence of subtasks and whether the subject had a reason for the sequence, such as, “I will take the picture naming test first because I am good at it” (1 point for each specified sequence and 1 point for each reason); and (3) the time planned to be utilized for each subset (1 point for each subset). There was no upper limit of the score awarded for intention formation.

##### Intention retention phase

After the digit span forward and backward subscales from the Wechsler Adult Intelligence Scale, and the working memory test (Kliegel et al., 2005), the participants were required to recall their plan to perform the six subtasks. Then they completed the Stroop Test.

##### Intention initiation phase

The participants filled a personal information questionnaire, in which there was an item about their date of birth. After finishing this question, the participants initiated the six-element task on their own. If they completed the questionnaire without remembering to perform the six subtasks, the examiner would give them a hint (“Is there anything to do after having answered the question about your date of birth?”). If the subject still did not remember the task, the examiner told the participant to open the drawer, take out the material, and initiate the six subtasks. However, only initiating the six subtasks immediately after finishing the cue question was considered to be the correct action. The score of performance for this phase was a dichotomous response of yes or no.

##### Intention execution phase

The subjects performed the six subtasks in 6 min. The task fidelity was recorded as being either right (the participants executed the subtasks exactly according to the original plan) or wrong (the subtasks were executed inconsistently with the plan). The ultimate intention execution score was determined by adding the scores for each subtask (modification 3).

#### Other cognitive tests

##### Digit span test (DST)

The DST from the Wechsler scale was used to measure attention and short-term memory (Wechsler, 1997). The examiner read a series of digits with increasing span size (3–12 in the forward subscale, and 2–10 in the backward subscale), and the participants were asked to repeat the numbers. Scores were calculated based on the maximum digit span size the subject could correctly recall in each subscale (for a maximum score of 22).

##### Working memory test

The test consisted of 12 blocks. Each block consisted of 3–6 pairs of math problems in a true/false format (e.g., read 4/2–1 = 1) followed by pronunciation of a word (such as school). After each block, the subjects were asked to recall the word (Turner and Engle, 1989). The score was calculated as the number of words omitted or mistaken (with a maximum score of 53). Lower scores represented better working memory.

##### Stroop test

The test was used to measure inhibition (Houx et al., 1993), and consisted of six blocks, with four rows in each block. Each row consisted of four characters of color names (red, yellow, blue, and green). The color characters were printed in mismatched colors. The participants were required to name the color in which of the character was printed, and not based on the meaning of the word. The number of wrong answers was used as the score. Lower scores reflect better performance.

### Statistical analysis

To explore the characteristics of the subjects, we used independent sample *t*-tests and χ^2^-tests for continuous and dichotomous variables (and Fisher's exact test if needed), respectively. For comparisons of intention formation and intention execution scores, linear regression models were constructed separately. The scores of intention formation and intention execution were added to a linear regression model as dependent variables, with disease status (PD or healthy) as independent variable, and the scores were adjusted for age, sex, and the scores of DST, working memory, and the Stroop test. For intention execution score, secondary analyses were carried out to further control for intention formation, intention retention, and fidelity. The logistic regression analyses were performed to analyze intention retention, intention initiation, and executive fidelity, with disease status (PD or healthy) as independent variables and controlling for age, sex, and the scores of DST, working memory, and the Stroop test. Spearman correlation was used to evaluate the association between clinical and demographic characteristics and PM performances of distinct phases. Data analyses were performed using SPSS 15 for Windows (SPSS Inc., Chicago, IL, USA). A *p* < 0.05 was regarded as statistically significant.

## Results

### Subjects' characteristics

The characteristics of the subjects are presented in Table 1. The demographic data (age, gender, and education), as well as MMSE score, were similar in the PD patient group and control group. The PD patients performed significantly worse relative to the healthy controls in the DST, working memory and Stroop tests. Although patients with major depressive disorder were excluded, HRSD scores were higher in the PD patients than the controls, despite the fact that all the PD patients' HRSD scores were ≤ 8 (Table 1).

**Table 1:** Clinical characteristics of PD patients and healthy controls.

	**PD, *n* = 31**	**HC, *n* = 27**	***p***
Age	66.42 ± 7.80	67.63 ± 8.14	0.566
Gender (male/female)	20/11	15/12	0.487
Education (years)	13.16 ± 3.14	13.30 ± 3.09	0.870
Disease duration (years)	5.32 ± 4.07		
**MEDICATION, n (%)**			
Amantadine	14 (45.2)		
Levodopa	24 (77.4)		
Receptor agonist	14 (45.2)		
Levodopa equivalent daily dose (mg)	457 ± 288		
UPDRS	41.74 ± 15.64		
H-Y stage	2.29 ± 0.60		
Side of onset (left/right)	14/17		
MMSE	28.55 ± 1.29	28.89 ± 0.93	0.260
HRSD	6.10 ± 2.0	1.89 ± 1.45	<0.001
DST	12.19 ± 2.12	13.41 ± 1.91	0.027
Working memory	12.06 ± 5.09	5.15 ± 3.36	<0.001
Stroop test	9.10 ± 4.61	2.15 ± 2.43	<0.001

*DST, Digit span test; HC, healthy controls; H-Y stage, Hoehn and Yahr Stage; MMSE, Mini-Mental State Examination; HRSD, Hamilton Rating Scale for Depression; PD, Parkinson's disease; UPDRS: unified Parkinson's disease rating scale*.

### PM examination

#### Intention formation phase

Before controlling for other cognitive functions, the score in intention formation was significantly lower in the PD patients (*p* < 0.001 vs. healthy controls; β = 0.701, 95% CI: 3.952–6.911, Table 2). After controlling for age, sex, DST, working memory and the Stroop test, the intention formation performance was still significantly worse in PD patients (*p* < 0.001 vs. healthy controls; β = 0.767, 95% CI: 3.765–8.124). We did not observe significant influences of DST, working memory, and Stroop test on intention formation.

**Table 2:** Regression analysis results for PM performances of PD patients and healthy controls.

	**PD, *n* = 31**	**HC, *n* = 27**	**β/OR**	**95% CI**	***p***
Intention formation	8.16 ± 2.62	13.59 ± 3.00	0.701	3.952–6.911	<0.001
Adjusted^*^			0.767	3.765–8.124	<0.001
Retention right, n (%)	25 (80.6)	24 (88.9)	0.521	0.117–2.322	0.392
Adjusted^*^			5.571	0.416–74.546	0.194
Initiation on time, n (%)	11 (35.5)	14 (51.9)	0.511	0.178–1.465	0.212
Adjusted^*^			1.905	0.274–13.261	0.515
Fidelity right, n (%)	27 (87.1)	25 (92.6)	0.540	0.091–3.210	0.498
Adjusted^*^			3.803	0.152–95.370	0.417
Execution score	177.74 ± 37.85	203.63 ± 26.87	0.368	8.375–43.400	0.004
Adjusted (model 1)^*^			0.013	−21.450–23.348	0.933
Adjusted (model 2)^**^			0.049	−23.170–30.011	0.797

HC, healthy controls

* *Adjusted for age, sex, and the scores of DST, working memory and Stroop test*.

** *Adjusted for age, sex, and the scores of DST, working memory, Stroop test, intention formation, intention retention and fidelity*.

#### Intention retention and intention initiation phases

A logistic regression model failed to show a statistically significant difference between PD patients and healthy controls in intention retention and intention initiation, regardless whether controlling for age, sex, DST, working memory and Stroop test results (Table 2). Results from the DST, working memory, and the Stroop test were not associated with intention retention and intention initiation.

#### Intention execution phase

Execution fidelity did not differ between PD patients and healthy controls, whether or not adjustments were made for age, sex, DST, working memory, and Stroop test (Table 2). Execution score was significantly lower in the PD patients than in the controls before adjusting for confounding factors (177.74 ± 37.85 vs. 203.63 ± 26.87, *p* = 0.004). However, adjusting for age, sex, DST, working memory, and Stroop test eliminated this difference. Further regression analysis that incorporated intention formation, intention retention, and fidelity also failed to show any difference in the execution score between the two groups. Working memory and fidelity were associated with the execution score (for working memory: β = −0.434, *p* = 0.015; for fidelity, β = −0.332, *p* = 0.026) (Table 2).

### Correlation analysis

The results of Spearman correlation analyses are shown in Table 3. Intention, executive fidelity, and executive score were correlated with disease duration and the Hoehn-Yahr stage. In addition, intention retention was correlated with disease duration.

**Table 3:** Correlation coefficients between clinical and demographical characteristics and PM performance in distinct phases.

	**Intention formation**	**Intention retention**	**Intention initiation**	**Intention executive fidelity**	**Intention execution score**
Age	−0.354	−0.064	0.163	0.194	−0.238
Disease duration	0.308	−0.612^***^	0.234	−0.434^*^	−0.524^**^
H-Y stage	0.004	−0.208	0.096	−0.371^*^	−0.575^***^
Education	−0.129	−0.009	0.145	0.253	0.221
HRSD	0.17	−0.183	0.227	−0.354	−0.285

*** p < 0.001;

** p < 0.01;

* p < 0.05.

*H-Y stage, Hoehn and Yahr Stage; HRSD, Hamilton Rating Scale for Depression. Values in bold indicate a statistically significant correlation (p < 0.05)*.

## Discussion

The current study showed impairments in the intention formation and execution phases of complex PM tasks in PD patients. However, a group difference in intention execution score was no longer evident after controlling for other cognitive tests, especially working memory. Furthermore, intention execution impairment was associated with disease severity and disease duration.

Earlier studies revealed impairment in the intention formation phase in PD patients (Kliegel et al., 2005). However, co-varying working memory reduced this group difference in the intention formation to almost non-significance (*p* = 0.05). Our results were partially inconsistent with this previous study and suggested that PD patients had significant impairment in intention formation even after controlling for other cognitive functions. This difference may be related to our alteration (modification 2) of the test protocol. Because of the paradigm design, 6 min was not enough to complete all the subtasks. In Kliegel's et al. (2005) study, the three types of subtasks were given equal weight, so it did not matter which type of the subtask was the final one (the one that might not be completed). The subjects' planning processes relied mainly on just remembering and implementing the rules. In the present study, the best strategy was to complete the subtasks weighted to be the most early-on in order to avoid not finishing it. Thus, our scoring system required better strategic planning than Kliegel's, and it therefore is more likely to find independent impairment in the intention formation phase in PD than the methods used in the prior study.

Our result found PD patients performed worse in intention execution, which is different from what was reported in Kliegel et al. (2005), possibly reflecting the third modification in the scoring of execution to make it more sensitive. After controlling for other cognitive abilities, group difference in intention execution became non-significant. Working memory and fidelity were associated with the score of intention execution. It is logical to suggest that the ability to follow the plan (fidelity) influences the intention execution. For working memory, previous studies have suggested it is related to PM performance of PD (Altgassen et al., 2007; Costa et al., 2008b). Disturbance of the frontostriatal circuit is critical for executive dysfunction and especially working memory in PD patients, because it represents a key substrate for intention execution of PM (Kehagia et al., 2010). Accordingly, we speculate that frontostriatal circuit dysfunction resulted in working memory deficits and intention execution impairment in the PD patients.

In the study of Kliegel et al. (2005) the PD group showed a trend toward intention initiation impairment. However, this result was not confirmed by our study with a larger sample. Some previous studies suggested impairment of self-initiation in event based PM in PD (Katai et al., 2003; Kliegel et al., 2005; Whittington et al., 2006), but other studies suggested that PD patients could initiate the event based PM tasks as well as healthy controls (Costa et al., 2008b; Raskin et al., 2011), especially when they preferentially focused on the PM task instead of the ongoing task (Altgassen et al., 2007) or used focal cues (Foster et al., 2009). They explained that the non-focal cues relied on a monitoring process, while focal cues induced a spontaneous reflective process; the former was an attention-demanding process, and relied more on executive function. In the current study, we used a focal cue, and this may partially explain why there was no difference in intention initiation between the two groups. Moreover, we carried out the PM task during the “ON” state in contrast to the “OFF” state in the study by Kliegel et al. (2005). The use of dopaminergic medications might well have improved the intention initiation performance in the present study. The effect of dopaminergic medication on prospective memory performance in PD remains unknown. One study using *de novo* PD patients revealed a marginal impairment in prospective memory (Pagni et al., 2011). Costa and colleagues used an “ON-OFF” comparison and showed that 200 mg levodopa could improve TBPM in PD (Costa et al., 2008a). However, also using an “ON-OFF” comparison, Foster et al. showed no significant effect of dopaminergic medications on the EBPM task, it is noteworthy that their “ON” state was defined as with the patients' regular anti-parkinsonian medications [including levodopa, dopamine agonists and catechol-O-methyl transferase (COMT) inhibitors] (Foster et al., 2009). The effects of levodopa and dopamine agonists on prospective memory in PD warrant further investigation.

Although the plan formed in our study was more complicated than most plans in previous PM studies, there was still no impairment in the intention retention in PD. This finding is in agreement with Kliegel's et al. (2005) findings. The intention retention component demands mainly retrospective memory storage capacity, which is associated with hippocampal functioning (Kliegel et al., 2011). Most studies on PM functions of PD revealed PM impairments in spite of well-preserved retrospective memory (Katai et al., 2003; Whittington et al., 2006; Altgassen et al., 2007; Costa et al., 2008a; Foster et al., 2009; Raskin et al., 2011). The evidence, including ours here, supports the view that retrospective memory ability is unlikely to be the main cause of PM impairment in PD. Thus, it may not be feasible to improve PM by focusing on ameliorating retrospective memory.

The current study found an association of intention execution impairment with disease duration and severity. This is consistent with the findings of Whittington et al. (2006). As the disease progresses, frontostriatal impairment worsens and more brain regions are involved (Braak et al., 2003). Therefore, it is reasonable to think that the performance of PM declines with increasing disease duration and severity.

There is great heterogeneity of cognitive impairment in PD. Lewy body pathological changes and Alzheimer-related pathological changes are two main causes, and in some patients vascular lesions may also contribute to cognitive impairment in PD (Svenningsson et al., 2012). The involved neurotransmitters include dopamine, acetylcholine, and norepinephrine (Kehagia et al., 2010). Therefore, executive function, visuospatial function, and memory are commonly undermined. Executive function and retrospective memory are essential for PM (Kliegel et al., 2011; Ramanan and Kumar, 2013). Although in the present study, impaired intention formation was not attributed to the attention, working memory, or inhibition functions which were tested. This dysfunction in intention formation might be explained by other aspects of executive function, as it is complex and encompass a series of abilities including inhibition, working memory, task shifting (cognitive flexibility), reasoning, problem solving, and planning, etc. (Diamond, 2013). A comprehensive executive function assessment in future studies might provide a better explanation. In addition, a prior study revealed retrospective memory impairment in PD and its association with PM dysfunction (Raskin et al., 2011). The preserved intention retention in the present study may be accounted for by a relatively good cognitive capability or a ceiling effect. Patients with PD may have varied cognitive abilities, from normal cognition, to amnestic and non-amnestic mild cognitive impairment, and dementia (Svenningsson et al., 2012). Costa and colleagues showed that the impairment of PM in PD depended on the global cognitive function (Costa et al., 2015). Future research focusing on specific patient groups would better reveal PM impairment in these groups.

There are some limitations to our study. First, the assessment of intention formation and intention execution yielded scores (continuous variables), while intention retention, initiation, and intention execution fidelity were described in terms of dichotomous variables. The latter thus may lack power to detect impairments. But in this kind of complex PM paradigm, it would be too complicated to add many task initiations like a simple PM paradigm such as in Katai et al.'s study (2003). This complex PM task is sensitive to impairments in intention formation and intention execution, whereas simple PM tasks are sensitive to impairments in intention initiation. Secondly, task switching is another important component of executive function in addition to working memory and inhibition, and plays a role in PM (Diamond, 2013; Costa et al., 2014). The present study did not utilize a test focusing on task switching. Thirdly, the present study only utilized neuropsychological tests; the lack of functional imaging modalities impeded further exploration of underlying mechanisms.

In summary, the intention formation phase was specially impaired in PD patients. The poorer performance of intention execution in PD was related to working memory deficits. In addition, PM impairment might progress with increasing disease duration and severity.

## Author contributions

Conception and design of the study: H-BC and WS. Recruiting the subjects and collecting demographic and clinical information: S-HJ, S-HL, and WS. Acquisition, analysis, and interpretation of data: KL and S-HJ. Drafting the manuscript: S-HJ, KL, and H-BC. All the other authors critically revised the draft and approved the final version.

### Conflict of interest statement

The authors declare that the research was conducted in the absence of any commercial or financial relationships that could be construed as a potential conflict of interest.
